# A Review of the Relationship Between EFL Teachers’ Academic Buoyancy, Ambiguity Tolerance, and Hopelessness

**DOI:** 10.3389/fpsyg.2022.831258

**Published:** 2022-03-01

**Authors:** Shuyun Huang

**Affiliations:** School of Foreign Languages, Huaiyin Normal University, Huai’an, China

**Keywords:** academic buoyancy, ambiguity tolerance, hopelessness, second/foreign language education, EFL teacher

## Abstract

Second/foreign language education has been approved emotionally tense due to its inherent challenges, adversities, complications, and ambiguities. These factors can affect various language teaching and learning domains. Hence, it is critical for EFL teachers to be buoyant and tolerant of ambiguity so that they can teach efficiently and prevent a sense of hopelessness that can damage everything. Although there are investigations on these variables in L2 contexts, their main focus has been on EFL students and teachers’ perspectives have been largely ignored. Against this shortcoming, this study aimed to review the definitions, conceptualizations, and research findings related to teachers’ academic buoyancy, ambiguity tolerance, and hopelessness. Moreover, practical implications for EFL teachers and teacher trainers are presented to increase their awareness of language teaching challenges and ways to overcome them. Finally, the study provides directions for future research.

## Introduction

Undoubtedly, teaching is one of the most difficult and emotionally intensive professions all around the world (Benevene et al., 2020). The inherent complications, twists, and turns in teaching occur in association with teachers’ emotions, psychological states, interpersonal skills, and varying pedagogical knowledge and practices. The toughness of teaching career increases in second/foreign language education due to the fact that L2 teachers have to deal with numerous factors and challenges at the same time (Li, 2021). Not only do they need sufficient pedagogical skills, but also an awareness of many psycho-emotional factors, (cross) linguistic disparities, and cultural issues that make teaching process a complex nested system with many intertwined layers (King and Ng, 2018). These concerns moved the field toward a shift of attention from negative emotions to positive emotions and their influential roles in various aspects of language teaching and learning (Derakhshan et al., 2019; Li and Yang, 2021; Xie and Derakhshan, 2021). This trend was first put in place by humanism and positive psychology both of which highlighted the effects of dwelling on positive emotions in generating many desired outcomes in academic contexts (Seligman, 2011; MacIntyre et al., 2019; Wang and Derakhshan, 2021b; Wang et al., 2021). However, they have never disregarded negativities, stressors, and challenges existing in education. Instead, they invest more time and effort on the positive side of life and the coming consequences of such a concentration (MacIntyre and Mercer, 2014).

Now, it is axiomatic that L2 education is a challenging task with many adversities and contradictory encounters. To survive and perform efficiently in such a demanding and highly accountable milieu, EFL teachers must strike a balance between their pedagogical expertise and psychological awareness of inner drives of instruction. This entails academic buoyancy which refers to the ability to manage, tolerate, and overcome the adversities and setbacks of an educational context (Comerford et al., 2015). As put by Zhang (2021), buoyancy is the positive side of resilience that is affected by both internal and external factors (e.g., inner feelings and contextual factors). Additionally, being buoyant in the face of adversities existing in academia is a precondition for transmitting knowledge on the part of the teachers. Research indicates that academic buoyancy can bring about different outcomes for EFL teachers and students such as increasing their motivation, engagement, self-esteem, confidence, self-efficacy, interpersonal skills, enjoyment, identity growth, agency, and the like (Putwain et al., 2015; Yun et al., 2018; Zhang, 2021). These findings signpost that despite adversities and setbacks, EFL teachers still can work their ways out and boom their performance and expertise. This is contingent upon their awareness and management of challenges and uncertainties overwhelming the career. There are many situations in L2 education that are inconsistent and diverse from the perspective of teachers’ native language and cultural norms. They must take such disparities and tough situations as opportunities and points of interest rather than hindrances. More technically, they must have *ambiguity tolerance* (AT, hereafter) to manage uncertainties and ambiguous encounters and move forward in their profession and just focus on their penultimate goal which is students’ learning and achievement.

The notion of AT is defined as one’s predilection to perceive ambiguous situations/stimuli as desirable (Budner, 1962). It is a psychological variable that shows how an individual deals with ambiguous events seeing them as neutral, threatening, or an opportunity. AT is regarded as a pivotal variable in one’s emotional and cognitive orientation toward his/her life and occupation (McLain et al., 2015). Research, in L2, has proved the potential and impact of AT on students’ mastery of different language skills and sub-skills, willingness to communicate (WTC), communicative competence, cultural adaptability, and intelligence, and weakens their anxiety (e.g., Atamanova and Bogomaz, 2014; Genç, 2016; Vahedi and Fatemi, 2016; Alahdadi and Ghanizadeh, 2017; Trabanco, 2017, unpublished^1^; Soodmand Afshar and Khasemy, 2019; Wang and Guan, 2020). As for teachers, AT has been found to affect their burnout level, emotional intelligence, professional practice, wellbeing, work engagement, satisfaction, and lowers their job stress (Hammond et al., 2017; Iannello et al., 2017; Zhaleh et al., 2018; Han and Wang, 2021). Nevertheless, the effect of buoyancy and AT on EFL teachers’ degree of hopelessness has been widely ignored in the pertinent literature. Hopelessness as a hindrance of desirable teaching performance occurs when EFL teachers feel dubious about the future of their profession. It can also drastically influence teaching-learning cycle, yet it has caught insufficient scholarly attention. Against this misgiving, the present article aims to review the body of knowledge in this domain and offers some trends for future scrutiny.

## Background

### The Concept of Buoyancy in Education

It is widely embraced that L2 education is a tense and challenging context to be in due to the adversities, conflicts, pressures, difficult work-loads, and linguistic-cultural disparities and inconsistencies inherent in it. This requires a positive mood to stay firm and resilient when facing challenges and failures in academia (Martin and Marsh, 2019). This concern led to the emergence of a new concept grounded in PP dubbed as *academic buoyancy* which refers to one’s ability to monitor, navigate, and cope with minor academic ups and downs in L2 education and progress toward success (Yun et al., 2018). To put it simply, it concerns one’s capacity to locate and take care of academic adversities and obstacles that occur in his/her academic life (Martin and Marsh, 2008). Therefore, it is a psychological variable that can be considered as a positive, fruitful, and adjustable response to daily academic challenges (Putwain et al., 2012). Like other psycho-emotional factors involved in second/foreign language education, academic buoyancy is mutable and dynamic and it is affected by both inner and outer drives to humans. That is to say, internal factors and personality traits as well as external-contextual factors influence the degree and nature of buoyancy (Comerford et al., 2015). In essence, academic buoyancy focuses on strengths than weaknesses and is more proactive when encountering challenges. Moreover, it explores “many and healthy” cases rather than extreme ones (Martin and Marsh, 2019). That is why, academic buoyancy is perceived as the positive version of resilience (Xue, 2021; Zhang, 2021).

### Cognates of Academic Buoyancy

The notion of academic buoyancy positioned itself in the literature and terminology of L2 education after the seminal work of Martin and Marsh (2008) who first defined and laid the stepping stones of the construct in language learning. A decade over such a breakthrough, many terms have been projected and used synonymously with buoyancy. Although they seem similar on the face of it, they have different scopes, denotations, and functions. Four highly cited cognate terms are *resilience*, *hardiness, coping,* and *immunity*. Resilience is a broad concept that concerns general difficulties that an individual faces. It does not fit with the setbacks that frequently happen in academic settings as it only examines a small and extreme group of cases (Martin and Marsh, 2019). Another analogous concept is hardiness which pertains to one’s ability to fight against and reduce the negative impacts of stress on his/her performance and practice (Hiver and Dörnyei, 2017). The next parallel construct is coping which describes a person’s various strategies to solve the problems and conflicts or alter how they are perceived (Somerfield and McCrae, 2000). Hence, they are specific techniques employed to tackle an aversive situation. The last cognate here is immunity which has its roots in biology and medical sciences. Simply, it is the defensive mechanisms or armoring systems that a person utilizes to diminish and stop encountering adversities, turbulences, and damages to his/her behaviors and practice (Hiver, 2017). The unique characteristic of immunity is that it is extemporaneous, unplanned, and double-edged in that it can cause positive and negative outcomes at the same time (Hiver, 2017).

### The Notion of Ambiguity Tolerance

The concept of ambiguity tolerance (AT) has been the focus of research in different fields such as psychology, sociology, and education for more than 70 years. Correspondingly, various definitions and interpretations have been offered for the concept. One of the most common definitions was delivered by Furnham (1994) who described AT as the way an individual (or a group) perceives and copes with the information about ambiguous situations/events full of new, complex, or incompatible cues. For Brown (2000), it is the extent to which one is cognitively eager to tolerate ideas that are contrary to his/her own belief system or knowledge base. In language education, AT is the ability to courageously face new ambiguous situations without feeling frustrated (Ellis, 1994). As put by Furnham and Marks (2013), this construct influences different aspects of human’s emotional and cognitive functioning, cognitive style, belief, value, and attitude systems, interpersonal skills, and problem-solving. AT has been used interchangeably with intolerance of ambiguity, (in)tolerance of uncertainty, uncertainty avoidance, and uncertainty management. While drawing a rigid boundary around these concepts is almost impossible, it can be claimed that AT differs from these similar terms in that it is a sort of grudging acceptance of a new situation. In other words, it focuses on the very unfamiliar stimuli, while intolerance of ambiguity or uncertainty cares about one’s psychological reaction/response to the stimuli that is mostly seen as a potential threat.

### Ambiguity Tolerance in Language Education

Due to its complex nature and ever-present challenges and adversities, language education has been one of the most fertile grounds to spread the seeds of AT. Second/foreign language learning and teaching are both emotionally tense. Hence, in a context laden with disparities and unfamiliar situations as per linguistic input and cultural norms, EFL teachers and learners need an awareness and practical skill to deal with ambiguities and conflicts (Abbe et al., 2007). Otherwise, they easily get trapped in linguistic, (inter)cultural, and sociocultural issues and get anxious, stressed out, confused, uncomfortable, and perform weakly (Genç, 2016). AT can be both a facilitator and a blocker of language teaching and learning depending on one’s ability to manage it (Kamran, 2011). Knowing this, different scholars in L2 contexts have done studies on various impacts of AT on teachers. The existing body of research shows that AT has positive associations with EFL teachers’ pedagogical practice, emotional intelligence, professionalism, wellbeing, work engagement, and job satisfaction and it reduces their job stress and burnout (Hammond et al., 2017; Iannello et al., 2017; Zhaleh et al., 2018; Wang and Derakhshan, 2021a). Moreover, it has been widely identified that augmented EFL students’ degree of AT affects their achievement in various tests and language skills/sub-skills (Khajeh, 2002, unpublished^2^; Başöz, 2015; Genç, 2016). Additionally, AT was approved to influence students’ WTC, communicative competence, cultural adaptability, and intelligence, and lowers their stress and anxiety (e.g., Atamanova and Bogomaz, 2014; Alahdadi and Ghanizadeh, 2017; Trabanco, 2017, unpublished (see footnote 1); Soodmand Afshar and Khasemy, 2019). Despite these investigations, researching AT in relation to EFL teachers and their psycho-emotional variables is still in its early stages that call for further explorations.

### The Conceptualization of Hopelessness

As stated earlier, L2 education is one of the most demanding tasks in the world as it is tied to one’s inner states and emotions. To perform well and guide the ship toward a calm and peaceful beach, EFL students and teachers must stay positive and be hopeful throughout the long journey. However, in reality, the process goes wrong and some teachers and students lose their hope and passion to go ahead. This lack of hope, passion, interest, and optimism led to the introduction of a new psychological variable in education called “hopelessness.” The term has mostly been used in clinical studies focusing on suicide and its antecedents. Therefore, in education, the variable has been defined sparsely due to its scan literature. Yet, the term is regarded as a negative feeling and expectation of one’s future emerging from his/her attributional styles and experiences (Rice et al., 2006; Yenilmez, 2010). It is a subjective emotion in which the person has lost his/her hope and faced many unsolvable dilemmas. This type of future pessimism can damage one’s confidence, control, motivation, passion, and courage (Pekrun et al., 2009). Hopelessness differs from depression and burnout on the basis of degree and context. Hopelessness is a lighter emotional problem that can be recovered and solved *via* appropriate strategies, while depression is a more acute psychological diagnosis that takes time to be solved (if ever). Moreover, burnout is more a work-related emotional exhaustion, while hopelessness is context-free and can occur irrespective of circumstances (Koutsimani et al., 2019). Despite the efforts, the concept of hopelessness in L2 education is still a murky one that needs complementary studies to firmly position itself and reach a solid conceptualization.

### The Sources, Consequences, and Solutions of Hopelessness

Hopelessness, as a damaging emotion in education, has different sources including self, family, society, economy, the school/work atmosphere, the staff, colleagues, and the materials and facilities used in education. All these sources are very critical in preventing or shaping hopelessness and pessimism in teachers. If left untreated, hopelessness can destroy everything in educational contexts. Research reveals that hopelessness can lead to stress, tension, social separation, identity crisis, anger, boredom, depression, anxiety, isolation, shyness, shame, and even suicide (Rice et al., 2006; Ismail, 2015; Lew et al., 2019). In a similar manner, many positive outcomes in academia depend on the removal and absence of hopelessness, such as high motivation for work, engagement, psychological wellbeing, academic success, job satisfaction, commitment, enjoyment, self-efficacy, self-esteem, perfectionism, and many more. These objectives are only obtainable through a loving and democratic teaching climate where EFL teachers know the existing challenges and ambiguities of the field and are able to take proper actions to cope with them and perform their responsibility perfectly. As hopelessness is made from various sources, a pedagogy of love together with emotion-oriented education can function as a curing drug.

## Implications, Research Gaps, and Future Directions

The present mini-review study was an attempt to shed light on the preventive role of academic buoyancy and AT of EFL teachers considering their hopelessness. It was argued that hopelessness is a damaging feeling that can end in dire consequences, such as burnout, poor performance, and depression in EFL teachers. Hence, it is essential to raise their knowledge and expertise regarding the innate challenges, adversities, and ambiguities involved in L2 teaching and learning. As a result, this study can be beneficial for EFL teachers in the sense that they can get to know the ups and downs of their profession and devise appropriate pedagogical strategies and techniques to perform well and stop the formation of negative emotions. Teacher trainers, also, can use this article to propose training workshops and seminars to improve EFL teachers’ awareness of L2 education challenges and the ways through which they can remove and solve negative factors of teaching and learning. Moreover, they can offer professional development programs on EFL teachers’ knowledge of emotions in education and how to deal with them in the class. As academic buoyancy, AT, and hopelessness can change over time and proper training, presenting novel and useful techniques to EFL teachers can make an outstanding change in these regards. Additionally, policy-makers at the macro level of education can use this study to revisit their planning and implementation of curriculums to place emotions in the center of all decisions as negative stressors like hopelessness can demolish all other aspects of education. L2 researchers are the last party to benefit from the ideas proposed in this article in that they can scrutinize this line of research focusing on the existing gaps. As stated in the background, most of the studies done on buoyancy and AT are based on self-reported data gleaned *via* surveys from EFL students and teachers’ views are largely ignored. This is due to the nature of these psycho-emotional variables whose measurement cannot be direct. Hence, most researchers utilize one-shot questionnaires/scales despite the fact that operational measures like diaries and journals can provide deeper insights. As for hopelessness, the available literature is mostly about the causes and results of students’ hopelessness and teachers’ perspectives are totally ignored. The reason behind this might be the misconception that hopelessness is more a student-related variable. Furthermore, avid researchers can run similar studies focusing on the role of cultural contexts on the variables discussed in this review. Another shortcoming of this line of research is that the three variables are time-sensitive and running longitudinal investigations can disclose their developmental trajectories better. Experimental studies on the use of specific methods to reduce or delete hopelessness in EFL teachers are also suggested to future researchers. Finally, hopelessness and AT that are less explored in positive psychology trend, in comparison with buoyancy, can be studied along with other variables like interpersonal communication skills, love, resilience, work engagement, organization structure/culture, care, self-efficacy, and the like. These propositions show that despite insightful findings researching buoyancy, AT, and hopelessness of EFL teachers still needs complementary studies using different research instruments and designs.

## Author Contributions

The author confirms being the sole contributor of this work and has approved it for publication.

## Conflict of Interest

The author declares that the research was conducted in the absence of any commercial or financial relationships that could be construed as a potential conflict of interest.

## Publisher’s Note

All claims expressed in this article are solely those of the authors and do not necessarily represent those of their affiliated organizations, or those of the publisher, the editors and the reviewers. Any product that may be evaluated in this article, or claim that may be made by its manufacturer, is not guaranteed or endorsed by the publisher.

## Footnotes

^1^Khajeh, A. (2002). The relationship between tolerance of ambiguity, gender and level of proficiency and use of second language learning strategies. Unpublished MA thesis. Tehran, Iran: Tarbiat Modarres University.

^2^Trabanco, A. F. (2017). Tolerance of ambiguity and L2 listening comprehension. Unpublished MA Thesis. Spain: University of Delaware.
